# Vaginal *Lactobacillus paragasseri* may attenuate fluconazole-resistant phenotypes in the subset of *Candida albicans* isolates and shows potential as a probiotic adjunct in antifungal therapy

**DOI:** 10.3389/fmicb.2026.1951876

**Published:** 2026-10-02

**Authors:** Qiuyue Wang, Hua Wang, Huirong Pan, Zhongyan Xu, Qi Cui, Huihai Zhao, Keran Jia, Fukun Wang

**Affiliations:** Clinical Laboratory, Bethune International Peace Hospital, Shijiazhuang, Hebei, China

**Keywords:** biofilm, *Candida albicans*, efflux pump, fluconazole-resistance, *Lactobacillus paragasseri*, probiotic therapy, vulvovaginal candidiasis

## Abstract

**Introduction:**

*Candida albicans* is the predominant etiological agent of vulvovaginal candidiasis (VVC). Fluconazole (FLU), a first-line azole antifungal agent, has encountered growing resistance among vaginal isolates of *C. albicans* due to prolonged, high-dose clinical administration. The development of such drug resistance arises from the synergistic interplay of multiple factors, with key mechanisms including enhanced biofilm formation, overexpression of drug efflux pump genes, and structural alterations in target enzymes driven by mutations in the *ERG11* gene. *Lactobacilli*, the dominant commensal probiotics in the vaginal microbiota, are known to inhibit biofilm formation by *C. albicans*, a process closely linked to the development of antifungal resistance. Consequently, vaginal lactobacilli may serve as a pharmacological adjuvant to enhance the antifungal efficacy of fluconazole against *C. albicans*. While existing research has primarily focused on the inhibitory effects of *Lactobacilli* on *C. albicans* proliferation, investigations into their impact on antifungal resistance remain limited.

**Methods:**

We investigate the impact of vaginal *L. paragasseri* on fluconazole-resistance phenotypes in a subset of clinical fluconazole-resistant *C. albicans* isolates. Guided by established fluconazole-resistance mechanisms, we first screened fluconazole resistant *C. albicans* isolates by assessing biofilm formation capacity, drug efflux pump gene expression, and *ERG11* gene mutations and mRNA expression. Next, analysis of the vaginal *Lactobacillus* composition in VVC patients and healthy controls showed a nominal, non-significant trend toward lower *L. paragasseri* detection in VVC-affected women. Motivated by this trend and by prior evidence that *Lactobacilli* can modulate antifungal-resistance phenotypes, we examined whether *L. paragasseri* modulates fluconazole-resistance in clinically derived *C. albicans* isolates through functional assays. Subsequent functional assessments, including standard antifungal susceptibility testing, biofilm formation assays, and drug efflux pump gene expression analyses.

**Results:**

Vaginal *L. paragasseri* can attenuate fluconazole-resistant phenotypes in the tested subset of clinical fluconazole-resistant *C. albicans* isolates. In a murine vulvovaginal candidiasis model, adjunctive treatment with *L. paragasseri* provided adjuvant benefit when added to fluconazole therapy, reducing vaginal *C. albicans* colonization and alleviating infection-associated tissue damage. These in-vivo observations constitute preliminary pre-clinical evidence.

**Discussion:**

These findings demonstrate that vaginal L. paragasseri holds promising potential as a probiotic adjunctive therapy for *C. albicans*-induced VVC, offering novel insights for the development of alternative antifungal therapeutic strategies.

## Graphical abstract

**Figure:**
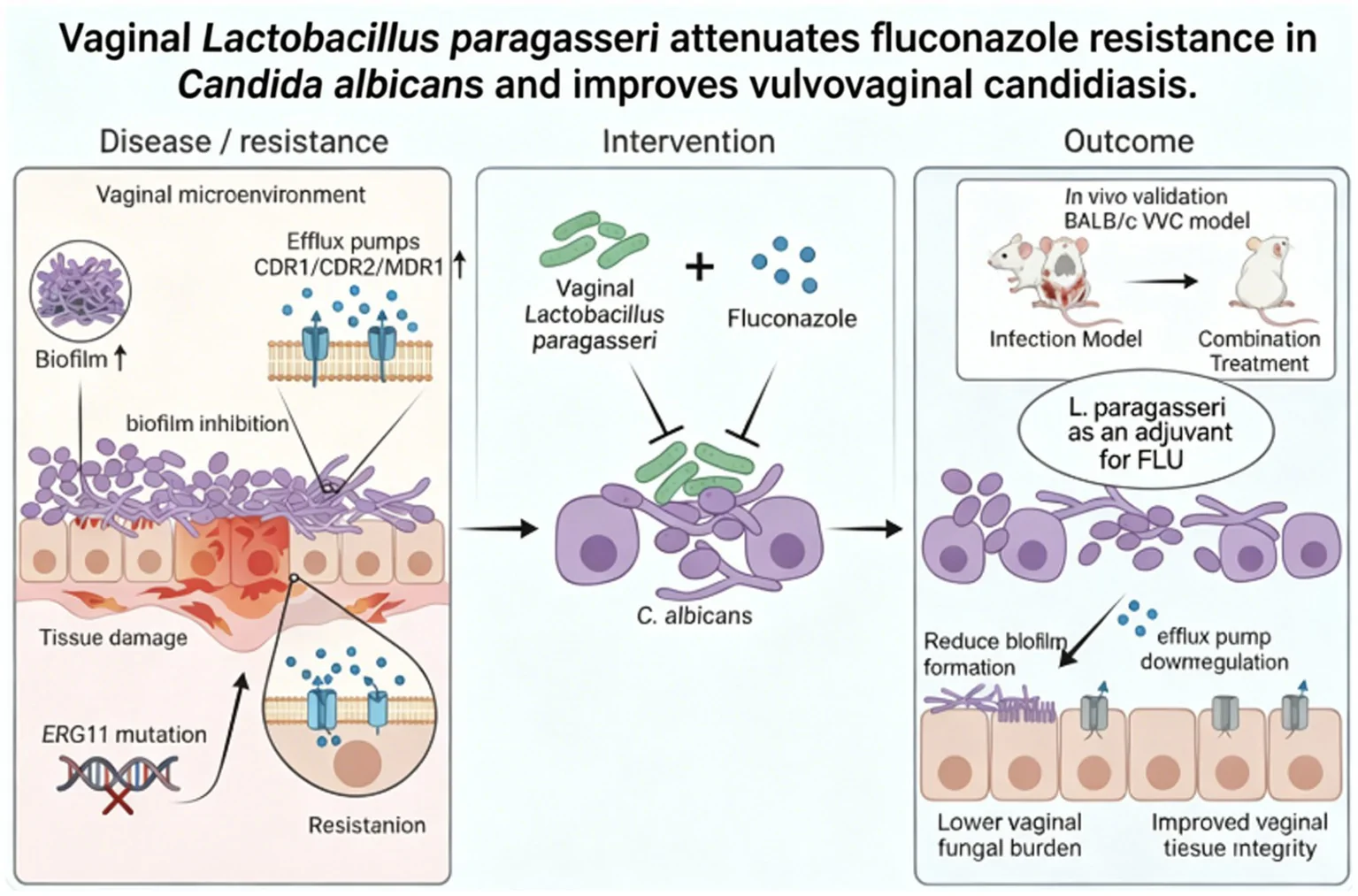

## 1 Introduction

*Candida albicans* is a ubiquitous opportunistic fungal pathogen that commonly colonizes the human oral cavity, gastrointestinal tract, and vaginal mucosa. Upon impairment of host immune function or disruption of local microecological homeostasis, *C. albicans* can transition from commensal colonization to pathogenic invasion, causing disorders including oral thrush, candidal enteritis, and vulvovaginal candidiasis (VVC). In severe cases, hematogenous dissemination may lead to life-threatening candidemia. VVC ranks among the most prevalent infectious diseases affecting women globally, with well-established risk factors including oral-contraceptive use, prolonged antibiotic exposure, diabetes mellitus, and genetic susceptibility. Epidemiological data demonstrate that approximately 75% of women will experience at least one VVC episode during their reproductive years, with 40–45% of affected individuals developing recurrent vulvovaginal candidiasis (RVVC) (Church et al., 2024; Jaeger et al., 2019). Moreover, VVC is particularly common during pregnancy, and severe or untreated infections may adversely affect maternal and pregnancy outcomes. Epidemiological studies have associated maternal VVC with low birth weight and an increased risk of preterm birth. These observations underscore the clinical importance of preventing and effectively managing VVC during pregnancy (Sule-Odu et al., 2020; Fu et al., 2016).

Currently, first-line clinical management of VVC primarily involves pharmacotherapy with single or combined antifungal agents (Surapaneni et al., 2021). Representative agents include systemic azoles (e.g., fluconazole, itraconazole) and topical azoles (e.g., clotrimazole, miconazole). Among these, fluconazole is a cornerstone first-line agent due to its favorable efficacy profile, broad accessibility, and convenience of oral administration. *Candida albicans* remains the predominant etiological agent responsible for approximately 80–90% of VVC cases (Umamaheshwari and Sumana, 2023; Zhao et al., 2020; Bhosale et al., 2025). In recent years, however, widespread and sometimes inappropriate clinical use of azole antifungals has led to a steady increase in azole resistance among vaginal *C. albicans* isolates (Sobel, 2023). Data from national antifungal resistance surveillance programs in China indicate that *C. albicans* accounts for 44.6% of clinically isolated yeast species, with 11.7% of these strains exhibiting resistance to triazole agents, most notably fluconazole (Wang et al., 2022). These trends highlight critical limitations in current therapeutic strategies, underscoring an urgent need for novel antifungal agents and innovative treatment paradigms to address azole-resistant VVC.

The principal mechanisms underlying azole resistance in *Candida albicans* include structural alterations in the drug target, upregulation of drug efflux pumps, and biofilm formation. *ERG11* encodes lanosterol 14α-demethylase, a key enzyme in the ergosterol biosynthetic pathway; ergosterol is an essential component of the *C. albicans* cell membrane. Specific missense mutations in *ERG11*, including Y132F/Y132H, K143R, and F126L, may alter the structure or drug-binding properties of lanosterol 14α-demethylase, thereby reducing azole binding and contributing to resistance (Lee et al., 2021; Ceballos-Garzon et al., 2023). The zinc cluster transcription factors Tac1, Mrr1, and Upc2 are important regulators of azole resistance in *C. albicans* (Nishimoto et al., 2019). Tac1- and Mrr1-mediated upregulation of the efflux pump genes *CDR1, CDR2*, and *MDR1* increases drug efflux and lowers the intracellular concentration of azole agents (Coste et al., 2006; Morschhäuser et al., 2007). In addition, biofilm formation can reduce azole susceptibility through limited drug penetration, extracellular matrix-mediated sequestration, and biofilm-associated changes in cellular physiology and gene expression, thereby promoting antifungal tolerance in *C. albicans* (Shen et al., 2025; Jiang et al., 2020).

As the dominant components of the vaginal microbiota, *lactobacilli* exert multifaceted biocontrol effects against *Candida* species, including nutrient and adhesion site competition, production of short-chain fatty acids (e.g., lactic acid, acetic acid) and subsequent pH reduction, direct inhibition of fungal proliferation, and induction of fungal cell apoptosis or necrosis (Er et al., 2019; Ene et al., 2013; Parolin et al., 2015). Notably, *Lactobacilli* species and strains exhibit distinct mechanisms of antagonism against *Candida*. For instance, metabolites produced by *Lactobacillus fermentum* and *Lactobacillus curvatus* inhibit *C. albicans* proliferation and reduce vaginal mucosal damage in murine VVC infection models (Jang et al., 2019); *Lactobacillus curvatus* and its secreted metabolites competitively inhibit Candida adhesion to vaginal epithelial cells (De Gregorio et al., 2020); *Lactobacillus rhamnosus* secretes the peptidoglycan hydrolase Msp1, which degrades chitin, a key component of Candida hyphal cell walls, thereby suppressing hyphal morphogenesis and virulence traits (Allonsius et al., 2019). Critically, the antagonistic activity of lactobacilli against *Candida* is highly strain-specific, with individual isolates displaying unique inhibitory mechanisms (MacAlpine et al., 2024). Furthermore, multiple lactobacilli strains have been shown to inhibit *C. albicans* biofilm formation (Takano et al., 2023; Rather et al., 2023). *C. albicans* biofilm formation is strongly associated with RVVC pathogenesis and the emergence of antifungal drug resistance (McKloud et al., 2021). Of note, the genus *Lactobacillus* underwent a major taxonomic reclassification in 2020; nevertheless, we retain the traditional nomenclature *Lactobacillus paragasseri* for consistency with most existing vaginal microbiology literature.

In recent years, the role of microbial ecology in shaping antimicrobial resistance has attracted increasing attention. Studies have demonstrated an association between gut microbiota composition and the abundance and distribution of antibiotic resistance genes (Feng et al., 2018). Moreover, probiotic administration has been reported to reduce the abundance of drug-resistant Enterobacteriaceae in the gut (Langdon et al., 2021). In addition, a cell-free extract of *Lactobacillus rhamnosus* (*L. rhamnosus* CFE) was shown to significantly inhibit drug efflux pump expression in *C. albicans* and reduce its efflux capacity in a dose-dependent manner (Dube et al., 2020). These findings provide important evidence that Lactobacillus may modulate the drug-resistant phenotype of *C. albicans*.

In summary, vulvovaginal candidiasis (VVC) remains highly prevalent globally. With the widespread long-term use of azole antifungals, *C. albicans* has increasingly developed resistance, presenting significant limitations to traditional antifungal therapeutic approaches and underscoring the urgent need for novel treatment strategies. As core beneficial members of the vaginal microbiota, lactobacilli exert competitive and antagonistic effects against *C. albicans* growth, providing a theoretical foundation for lactobacillus-based biotherapeutic interventions. In parallel with probiotic-based approaches, recent studies have explored the development of small-molecule efflux pump inhibitors that directly target *CDR1*, *CDR2*, and *MDR1* to restore azole susceptibility in resistant *C. albicans* isolates (Yehia et al., 2025; Gomaa et al., 2024). These strategies, together with Lactobacillus-mediated modulation, highlight the growing interest in adjunctive therapies that circumvent conventional resistance mechanisms. However, the precise mechanisms underlying lactobacilli-mediated effects on *C. albicans* remain incompletely elucidated. In our own case–control comparison, *L. paragasseri* showed a directional, though non-significant, trend toward lower detection in VVC specimens. Together with prior evidence that lactobacilli can modulate antifungal-resistance phenotypes of *C. albicans*, this observation prompted us to ask whether *L. paragasseri* can attenuate fluconazole resistance in clinical isolates. Accordingly, this study investigated the effects of *L. paragasseri* on drug sensitivity in the subset of clinical fluconazole-resistant *C. albicans* isolates, exploring its underlying mechanisms with a focus on biofilm formation and drug efflux-pump expression, and further assessed these effects in a murine VVC model. This work provides experimental data linking vaginal lactobacilli to *C. albicans* resistance and may inform adjunctive strategies for fluconazole-resistant VVC.

## 2 Materials and methods

### 2.1 Strains, growth conditions, and reagents

All clinical strains used in this study were isolated from vaginal secretion specimens collected at Bethune International Peace Hospital. A case–control design was applied, consisting of 50 women with VVC (case group) and 30 healthy female volunteers (control group). Identical vaginal swab sampling procedures were used for both groups. Inclusion criteria for the VVC case group: (1) Clinical diagnosis of vulvovaginal candidiasis with fungal positivity; (2) No antibiotics that may disrupt vaginal microbiota within 3 days prior to sampling; (3) Non-menstrual status (typically 3–5 days after the last menstrual period, or confirmed absence of menstruation at sampling); (4) Age ≥ 18 years. Exclusion criteria for the VVC case group: (1) Menstruation or active vaginal bleeding at sampling; (2) Pregnancy; (3) Systemic administration of antibiotics or antifungal agents within 3 days before sample collection. Inclusion criteria for healthy controls: (1) Age ≥ 18 years; (2) Non-menstrual phase at sampling; (3) No use of hormonal contraceptives within the past 3 months; (4) No antibiotic or antifungal treatment within the preceding 3 days. Exclusion criteria for healthy controls: (1) Menstruation or active vaginal bleeding; (2) Pregnancy; (3) Clinical symptoms suggestive of any form of vaginitis; (4) Positive fungal smear or fungal culture from vaginal swabs. *Candida albicans* strains were routinely cultured on yeast peptone dextrose (YPD) agar at 35 °C, while lactobacilli were cultured on de Man, Rogosa, and Sharpe (MRS) agar under the same temperature conditions. Multiple *L. paragasseri* strains were recovered from clinical vaginal specimens during strain screening. One representative clinical isolate *L. paragasseri*LPV08 was selected and applied for all subsequent *in vitro* and in-vivo functional experiments in this study. For liquid culture, single colonies of C. albicans were aseptically inoculated into YPD broth using sterile loops and were incubated at 35 °C with shaking at 160 rpm for 12–16 h until reaching the mid-logarithmic growth phase. Single colonies of lactobacilli were aseptically inoculated into MRS broth and incubated statically overnight at 35 °C. Key experimental reagents included fluconazole (FLU, MedChemExpress), antibiotics (streptomycin, ampicillin, gentamicin, and kanamycin, Solarbio Technology Co., Ltd., Beijing, China), and 17β-estradiol benzoate (Yuanye Bio). All reagents were prepared and stored strictly following the manufacturers’ protocols. Prior to experimentation, reagents were diluted to working concentrations using sterile phosphate-buffered saline (PBS) or dimethyl sulfoxide (DMSO), depending on their solubility characteristics.

### 2.2 Determination of the minimum inhibitory concentration of fluconazole against *Candida albicans*

The *C. albicans* suspensions were adjusted to 1 × 10^6^ CFU/mL. The concentration of *L. paragasseri* during co-incubation 1 × 10^8^ CFU/mL. Co-culture was performed in RPMI 1640 medium supplemented with 25 mМ MES and adjusted to pH 6.0 with NaOH. The pH was measured at the start and end of incubation to confirm maintenance within the target range (pH 6.0 ± 0.5). Following incubation at 35 °C for 24 h, the culture was streaked onto YPD agar plates supplemented with chloramphenicol, and a single *C. albicans* colony was isolated for subsequent determination. The minimum inhibitory concentration (MIC) of fluconazole was determined using the MA120 system and its corresponding antifungal susceptibility plates (Zhuhai Meihua Medical Technology Co., Ltd.) by the broth microdilution method (Palma et al., 2024). According to the manufacturer’s technical documentation, the susceptibility testing results obtained with this system are consistent with the CLSI M60 standard. A single colony was selected and suspended in sterile saline, and the cell suspension was adjusted to a turbidity equivalent to 0.5 McFarland. The adjusted suspension was then inoculated into an antifungal susceptibility plate compatible with the MA120 system and preloaded with a gradient of fluconazole concentrations. Following incubation at 35 °C for 24 h, the MIC values were read using the MA120 fungal identification and antimicrobial susceptibility analysis system.

### 2.3 Spot assay

Co-cultivation was performed as described in Section 2.2. After co-incubation, *C. albicans* was re-isolated from the co-culture on chloramphenicol-containing YPD agar, and the purified *C. albicans* was used for the spot assay. Activating *C. albicans* in YPD liquid medium. The *C. albicans* suspensions, with or without treatment with *L. paragasseri*, were adjusted to 1 × 10^5^ CFU/mL. Each suspension was subjected to three successive 10-fold serial dilutions. Equal volumes of each dilution were dotted onto YPD agar plates containing a fluconazole concentration of 8 μg/mL, arranged from the highest to the lowest concentration (Alvarez-Rueda et al., 2016). The plates were incubated at 35 °C for 48 h, after which the growth of *C. albicans* was compared between the *L. paragasseri*-treated and untreated groups.

### 2.4 Biofilm formation assay

Co-cultivation was performed as described in Section 2.2. After co-incubation, *C. albicans* was re-isolated from the co-culture on chloramphenicol-containing YPD agar, and the purified *C. albicans* was used for the Biofilm formation Assay. Activating *C. albicans* in YPD liquid medium. Log-phase *C. albicans* cells, with or without treatment with *L. paragasseri*, were resuspended in RPMI 1640 medium and adjusted to an initial concentration of 1 × 10^6^ CFU/mL. The cell suspensions were then inoculated into polystyrene 96-well plates, with three technical replicates per sample, and incubated at 35 °C for 24 h. After incubation, the wells were gently washed with phosphate-buffered saline (PBS) to remove non-adherent cells. The adherent biofilms were fixed with 100% methanol and stained with 0.5% (w/v) crystal violet. The bound crystal violet was subsequently solubilized with 33% (v/v) ice-cold acetic acid at 37 °C (Özarslan et al., 2024). The optical density was measured at 595 nm using a microplate reader. After staining with crystal violet, the biofilm morphology was observed under an inverted optical microscope (Olympus CKX53) at 400× magnification. At least three randomly selected fields per well were collected to observe the hyphal length and density.

### 2.5 Real-time fluorescent quantitative reverse-transcription PCR

Co-cultivation was performed as described in Section 2.2. After co-incubation, *C. albicans* was re-isolated from the co-culture on chloramphenicol-containing YPD agar, and the purified *C. albicans* was used for the subsequent RT-qPCR analysis. Activating *C. albicans* in YPD liquid medium. The concentration of the *C. albicans* suspension was adjusted to 1 × 10^6^ CFU/mL. Total RNA was extracted using the Yeast RNA Rapid Extraction Kit (PolymerBio Biotechnology Co., Ltd., Beijing, China). First-strand complementary DNA (cDNA) was synthesized using TransScript First-Strand cDNA Synthesis SuperMix (Full Synthetic Biotechnology Co., Ltd., Beijing, China) according to the manufacturer’s instructions. Quantitative reverse-transcription PCR (RT-qPCR) was performed using 2 × HS SYBR Green qPCR Mix (SanShi Biotechnology Co., Ltd., Shijiazhuang, Hebei, China) in eight-well PCR strip tubes. Each 25-μL reaction mixture contained 1 μL of cDNA template, 0.4 μL each of the forward and reverse primers, 12.5 μL of qPCR mix, and nuclease-free water. The amplification protocol consisted of an initial denaturation at 95 °C for 5 min, followed by 40 cycles of denaturation at 95 °C for 5 s, annealing at 57 °C for 15 s, and extension at 72 °C for 30 s. ACT1 was used as the reference gene to normalize the expression levels of the target genes. Relative gene expression levels were calculated using the 2^-ΔΔCt^ method (Toyoda et al., 2004). Data are presented as the mean ± standard deviation (SD) from at least three biological replicates. The primer sequences used in this study are listed in Supplementary Table 1.

### 2.6 *ERG11* gene and 16S rRNA gene sequencing

All samples were submitted to Beijing Ruibo Xingke Biotechnology Co., Ltd. for full-length Sanger sequencing using an ABI 3730XL platform. Species assignment was performed by the company using BLASTn algorithm (v2.13.0) against the NCBI RefSeq microbial 16S rRNA gene database, which includes reference sequences for all known species within the genus *Lactobacillus*. The threshold for species-level identification was set at ≥99.5% sequence identity and ≥99% query coverage. To improve the resolution for closely related species, all clinical isolates preliminarily identified as *L. paragasseri* through the above pipeline were subjected to an independent re-identification step: sequences were independently re-analyzed using BLASTn against the public NCBI database, and were further aligned with reference strains *L. paragasseri* DSM 26459 and *L. gasseri* ATCC 33323 to confirm clustering with the *L. paragasseri* type strain. Moreover, all *L. paragasseri* strains used in functional experiments showed ≥99.7% 16S rRNA sequence similarity to the reference strain DSM 26459. For each vaginal specimen, the dominant cultivable *Lactobacillus* isolate was obtained by plating on MRS agar and identified to the species level by full-length 16S rRNA gene sequencing, as described above. The percentage reported for each species represents the proportion of subjects in a given group whose dominant cultivable *Lactobacillus* isolate belonged to that species (number of subjects with that species/total number of subjects in the group × 100%).

### 2.7 Animal experimental procedures

Seven-week-old female BALB/c mice (18–20 g) were purchased from Beijing Huafukang Biotechnology Co., Ltd. (Beijing, China) and used to establish a murine vulvovaginal candidiasis (VVC) infection model (Nabeta et al., 2022; Yang D. L. et al., 2018; Menon et al., 2021). Following acclimatization for 7 days, Randomization: Mice were randomly assigned to different treatment groups using a computer-generated random number table (*n* = 4 per group): (1) Healthy control group (treated with physiological saline), (2) Untreated infection model group, (3) Fluconazole monotherapy group, (4) Fluconazole plus *L. paragasseri* combination therapy group. Except for the healthy control group, mice in the other three groups were subcutaneously injected with 10 μL of 20 mg/mL estradiol benzoate (dissolved in soybean oil, equivalent to 0.2 mg per mouse) once daily for 3 consecutive days prior to *C. albicans* inoculation to induce persistent estrus. This treatment was continued once every 2 days until the end of the experiment. Mice in the healthy control group received a 20 μL vaginal injection of physiological saline, while mice in the other three groups were vaginally inoculated with 5 × 10^6^ CFU of Drug-Resistant *C. albicans* LAB-V-24-130 per mouse. After inoculation, mice were held in an inverted position for 3–5 min to prevent fluid leakage, and this procedure was repeated daily for 7 consecutive days. Following the 7-day inoculation period, mice in the fluconazole monotherapy group and combination therapy group received daily treatment for 7 consecutive days: fluconazole was administered orally at 20 mg/kg body weight, while mice in the combination therapy group additionally received a vaginal inoculation of *L. paragasseri*LPV08 (1 × 10^8^ CFU/mouse). Mice in the healthy control group and untreated infection model group received an equal volume of physiological saline via oral gavage as a vehicle control. Blinding: The investigator responsible for vaginal fungal burden quantification (CFU counting) and histopathological scoring was blinded to the group allocation throughout the experiments. All experimental procedures were conducted in accordance with the Regulations on the Administration of Laboratory Animals (China) and were approved by the Institutional Animal Care and Use Committee (IACUC) of Bethune International Peace Hospital (Approval No. 2024-KY-341).

### 2.8 Local vaginal signs and reproductive organ anatomy in mice

Twenty-four hours after the final treatment administration, mice in each group were humanely euthanized via cervical dislocation in accordance with institutional guidelines. Subsequently, mice were immersed in 75% (v/v) ethanol for 2 min to achieve surface disinfection. Aseptic dissection was performed under a sterile laminar flow hood to harvest the reproductive tract, including the vagina, cervix, uterus, and ovaries. The gross morphological appearance of reproductive organs (e.g., vaginal redness, swelling, exudate, uterine congestion) and evidence of infection were systematically observed, photographed, and documented for each mouse (Gao et al., 2016).

### 2.9 Vaginal lavage smear microscopy

Twenty-four hours following the 7-day treatment course, the vaginal cavity of each mouse was lavaged with 100 μL of sterile phosphate-buffered saline (PBS). A 10 μL aliquot of collected vaginal lavage fluid from each mouse was pipetted onto a glass slide, spread evenly, air-dried at room temperature, and heat-fixed by three sequential passes through an alcohol lamp flame. After cooling to room temperature, slides were subjected to Gram staining, and the morphology of *C. albicans* in vaginal secretions was observed under a light microscope (Mphande-Nyasulu et al., 2026).

### 2.10 Quantification of vaginal tissue fungal burden

Vaginal tissue samples harvested from each mouse were weighed, and homogenized in sterile phosphate-buffered saline (PBS) using an ultrasonic homogenizer to a final concentration of 40 mg tissue per mL of PBS. Serial 10-fold dilutions of homogenates were prepared in sterile PBS, and 100 μL aliquots of appropriate dilutions were spread onto yeast extract-peptone-dextrose (YPD) agar plates supplemented with ampicillin (100 μg/mL), kanamycin (50 μg/mL), and streptomycin (100 μg/mL) (Gao et al., 2016). Plates were then incubated aerobically at 35 °C for 24–48 h. After incubation, colony-forming units (CFU) were counted manually, and results were calculated as CFU per gram of vaginal tissue.

### 2.11 Histological analysis of vaginal tissues

Sterilely harvested vaginal tissues were fixed in 4% (w/v) paraformaldehyde solution (Biosharp, Shanghai, China) for 48 h at 4 °C. Tissues were then dehydrated through a graded ethanol series, embedded in paraffin wax, and sectioned into 4 μm-thick slices. Sections were stained with hematoxylin and eosin (HE) and examined under a light microscope for histological changes (Yang L. et al., 2018).

### 2.12 Statistical analysis

Quantitative data are presented as mean ± standard deviation (SD) from at least three independent biological replicates. The Shapiro–Wilk test and Levene’s test were used to assess data normality and homogeneity of variances, respectively. For comparisons between two independent groups, an unpaired two-tailed Student’s *t*-test was used for normally distributed data; otherwise, the Mann–Whitney *U* test was applied. For comparisons involving three or more groups, one-way analysis of variance (ANOVA) followed by Tukey’s *post hoc* test was performed to control the family-wise error rate across multiple pairwise comparisons. Statistical analyses and data visualization were conducted using GraphPad Prism version 8.0 (GraphPad Software, Inc., San Diego, CA, United States). A two-sided *p*-value < 0.05 was considered statistically significant.

## 3 Results

### 3.1 Screening and identification of fluconazole-resistant *Candida albicans* strains

A total of 50 clinical *C. albicans* isolates were collected and screened for fluconazole susceptibility by broth microdilution (Section 2.2; Table 1). Applying the CLSI M60 breakpoint (MIC ≥8 μg/mL), 11 isolates were classified as fluconazole-resistant, yielding a resistance prevalence of 22% in this cohort. To further validate the resistant phenotype, all isolates were streaked onto YPD agar plates supplemented with fluconazole (8 μg/mL). The 11 resistant isolates showed markedly greater colony formation on fluconazole-containing plates than the remaining 39 isolates (Figure 1 and Supplementary Figure 1), consistent with the broth microdilution results.

**Table 1:** MIC values of fluconazole against *Candida albicans*.

Strain	MIC (μg/mL)	Strain	MIC (μg/mL)
LAB-V-24-013	8	LAB-V-24-094	8
LAB-V-24-022	16	LAB-V-24-112	16
LAB-V-24-025	8	LAB-V-24-124	8
LAB-V-24-030	8	LAB-V-24-130	8
LAB-V-24-077	8	LAB-V-24-135	8
LAB-V-24-091	16		

**Figure 1:**
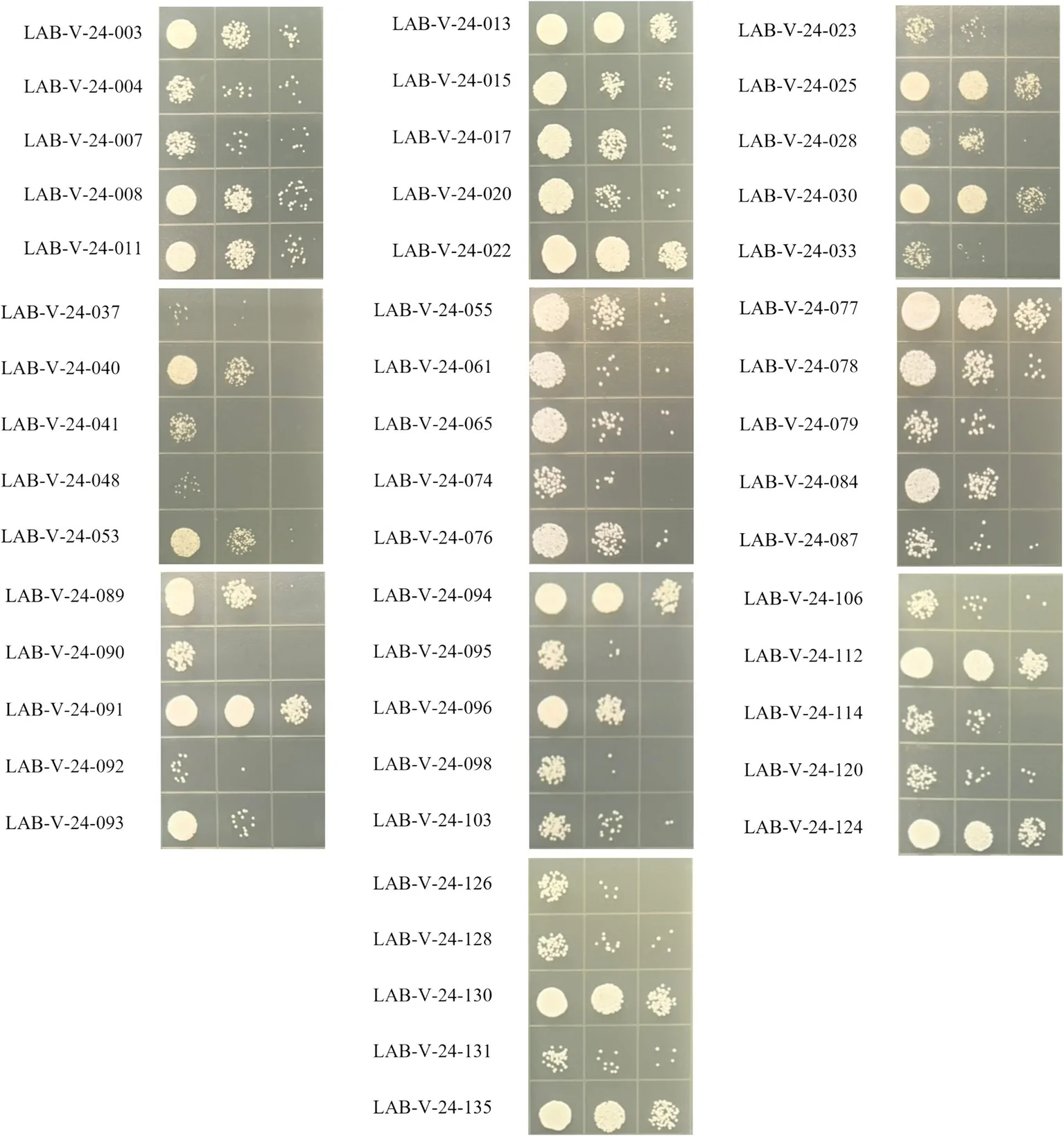
Determination of the fluconazole susceptibility phenotype of *C. albicans* by spot assay. *C. albicans* suspensions were adjusted to 1 × 10⁵ CFU/mL, followed by three successive 10-fold serial dilutions. The undiluted suspensions and serial dilutions were spotted from left to right onto YPD agar plates containing fluconazole (8 μg/mL) and incubated at 35 °C for 48 h.

### 3.2 Assessment of biofilm formation by *Candida albicans* using crystal violet staining

Biofilm formation by *Candida albicans* is closely associated with the transition from the yeast form to the hyphal form. The biofilm-forming capacity of the 11 fluconazole-resistant isolates identified above was evaluated using a crystal violet staining assay under biofilm-inducing conditions in RPMI 1640 supplemented with 10% fetal bovine serum (FBS). Biofilm biomass was quantified by measuring the optical density (OD) at 595 nm after crystal violet staining (Figure 2A). The results indicated that isolates LAB-V-24-022, LAB-V-24-025, LAB-V-24-112, and LAB-V-24-130 exhibited relatively high biofilm biomass and were classified as strong biofilm formers. Inverted microscopic examination revealed differences in biofilm morphology and cellular organization among the isolates. Isolates with strong biofilm-forming capacity displayed dense, interconnected biofilm structures, whereas isolates with weak biofilm formation predominantly exhibited yeast-form cells, with limited hyphal development (Figure 2B).

**Figure 2:**
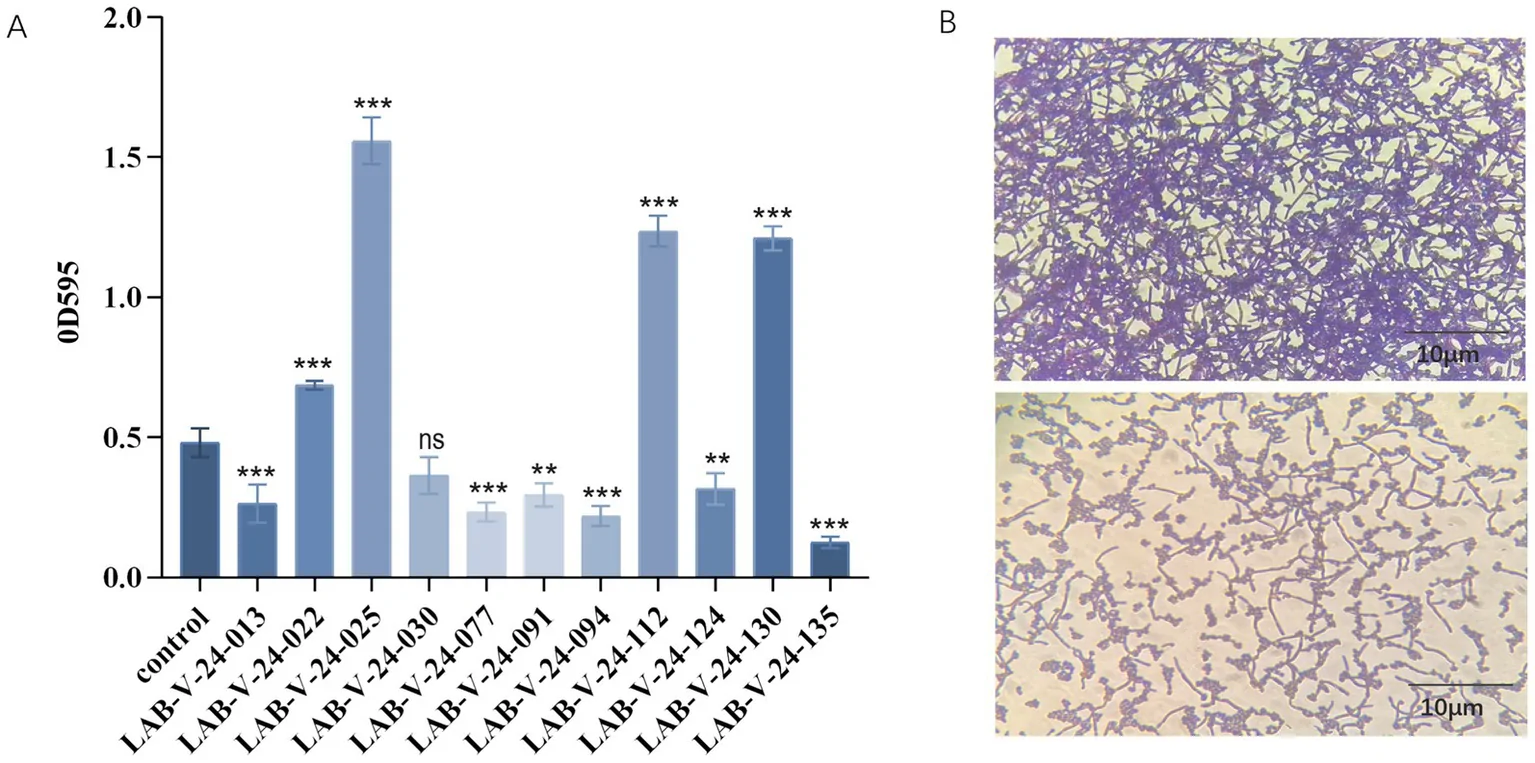
Assessment of biofilm formation by *Candida albicans* using crystal violet staining. **(A)** Compared with the reference strain SC5314, isolates LAB-V-24-022, LAB-V-24-025, LAB-V-24-112, and LAB-V-24-130 exhibited greater biofilm biomass. Data are presented as mean ± SD. *n* = 3 biological replicates per group. Statistical significance was determined by one-way ANOVA followed by Tukey’s *post hoc* test. ns, not significant; **p < 0.01, ***p < 0.001. **(B)** Inverted microscopic examination revealed that isolates with strong biofilm formation exhibited longer, densely intertwined hyphae, whereas isolates with weak biofilm formation displayed shorter and more sparsely distributed hyphae. The images are representative of three independent experiments.

### 3.3 RT-qPCR analysis of drug efflux pump-related gene expression in *C. albicans*

Drug efflux pumps contribute to antifungal resistance by exporting intracellular drugs and reducing their concentrations below the levels required to inhibit fungal growth in *C. albicans*. RT-qPCR was used to quantify the transcript levels of the drug efflux pump-related genes *CDR1, CDR2,* and *MDR1* in 11 fluconazole-resistant isolates. The expression levels of these genes varied among the isolates (Figure 3). Compared with the reference strain SC5314, isolates LAB-V-24-091, LAB-V-24-112, and LAB-V-24-130 exhibited higher expression levels of one or more efflux pump-related genes.

**Figure 3:**
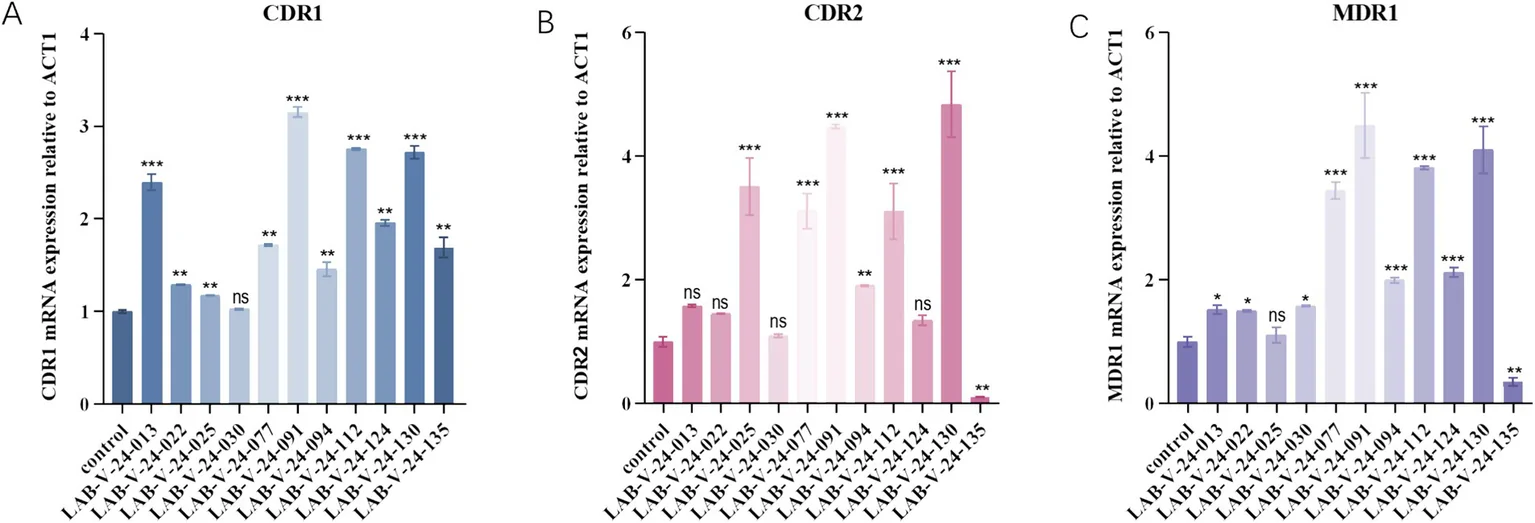
RT-qPCR analysis of drug efflux pump-related gene expression in *C. albicans*. **(A)** Compared with the reference strain SC5314, the expression levels of *CDR1* were higher in isolates LAB-V-24-091, 112 and 130. **(B)** Compared with the reference strain SC5314, the expression levels of *CDR2* were higher in isolates LAB-V-24-025, 077, 091, 112, and 130. **(C)** Compared with the reference strain SC5314, the expression levels of *MDR1* were higher in isolates LAB-V-24-077, 091, 094, 112, 124, and 130. Data are presented as mean ± SD. *n* = 3 biological replicates per group. Statistical significance was determined by one-way ANOVA followed by Tukey’s post-hoc test. ns, not significant; *p < 0.05, **p < 0.01, ***p < 0.001.

### 3.4 Sequencing of the *ERG11* gene to identify azole target-site mutations in *C. albicans*

Azole resistance in *C. albicans* can result from mutations in the *ERG11* gene, which encodes lanosterol 14α-demethylase (Erg11p), the target enzyme of azole antifungals and a key component of the ergosterol biosynthetic pathway. Missense mutations in *ERG11* may alter the structure of the Erg11p active site, thereby reducing the binding affinity of azole antifungals for the target enzyme. Consequently, azole-mediated inhibition of Erg11p may be reduced, contributing to the development of an azole-resistant phenotype. The *ERG11* gene was analyzed by Sanger sequencing, and the deduced amino acid sequences were aligned using SnapGene software. The results showed that isolates LAB-V-24-022, LAB-V-24-025, and LAB-V-24-091 carried the Y132H substitution, which corresponds to a nucleotide change from TAT to CAT (Table 2 and Supplementary Figure 2). Compared with the wild-type reference strain, the mutant isolates (harboring Y132H substitutions) showed upregulated *ERG11* mRNA levels, indicating that the identified resistance-associated point mutations may contribute to enhanced *ERG11* expression at the transcriptional level (Figure 4).

**Table 2:** Comparison of amino acid site mutations encoded by the *ERG11* gene.

Strain	Mutation site	Mutation situation	Base mutation information
LAB-V-24-022	Tyr132 → His	Tyr132 → His	TAT → CAT
LAB-V-24-025	Tyr132 → His	Tyr132 → His	TAT → CAT
LAB-V-24-091	Tyr132 → His	Tyr132 → His	TAT → CAT

**Figure 4:**
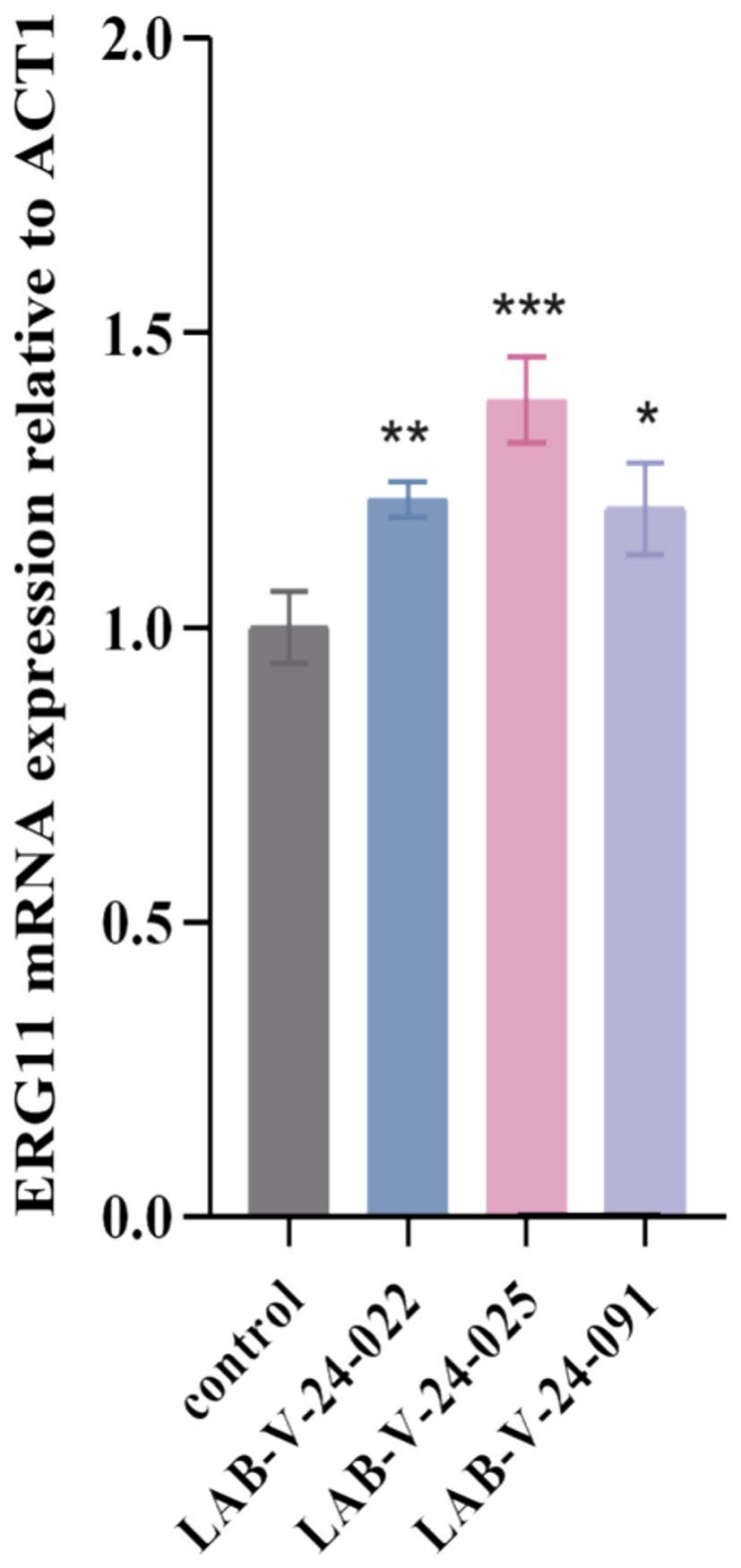
RT-qPCR analysis of *ERG11* gene expression in *Candida albicans*. Compared with the reference strain *SC5314*, the expression levels of *ERG11* in the isolates LAB-V-24-022, 025, and 091 were elevated to varying degrees. Data are presented as mean ± SD. *n* = 3 biological replicates per group. Statistical significance was determined by one-way ANOVA followed by Tukey’s *post hoc* test. *p < 0.05, **p < 0.01, ***p < 0.001.

### 3.5 Differences in the detection rate of dominant culturable *Lactobacillus* species between healthy and pathological specimens

This study included a total of 50 patients with VVC and 30 healthy controls. Statistical analysis was performed using Fisher’s exact test. *L. crispatus* was the dominant species in both groups (VVC 32/50, 64% vs. control 20/30, 67%; *p* = 0.81). *L. paragasseri* was recovered less frequently from VVC specimens than from controls (11/50, 22% vs. 10/30, 33%, *p* = 0.47), but this difference did not reach statistical significance. *L. gasseri* was detected only in VVC specimens (6/50, 12% vs. 0/30, *p* = 0.079), and *L. johnsonii* occurred in a single VVC isolate and was not tested (Figure 5). These percentages represent the proportion of subjects whose dominant cultivable *Lactobacillus* isolate belonged to each species. Importantly, changes in species-level detection rates should not be equated with functional outcomes, given the well-documented strain-specific functional heterogeneity of *Lactobacillus*. The directional trend toward lower *L. paragasseri* detection in VVC, together with the established role of vaginal *Lactobacilli* in antagonizing *C. albicans* and prior evidence that lactobacilli can modulate antifungal-resistance phenotypes, motivated the selection of *L. paragasseri* for the functional experiments below.

**Figure 5:**
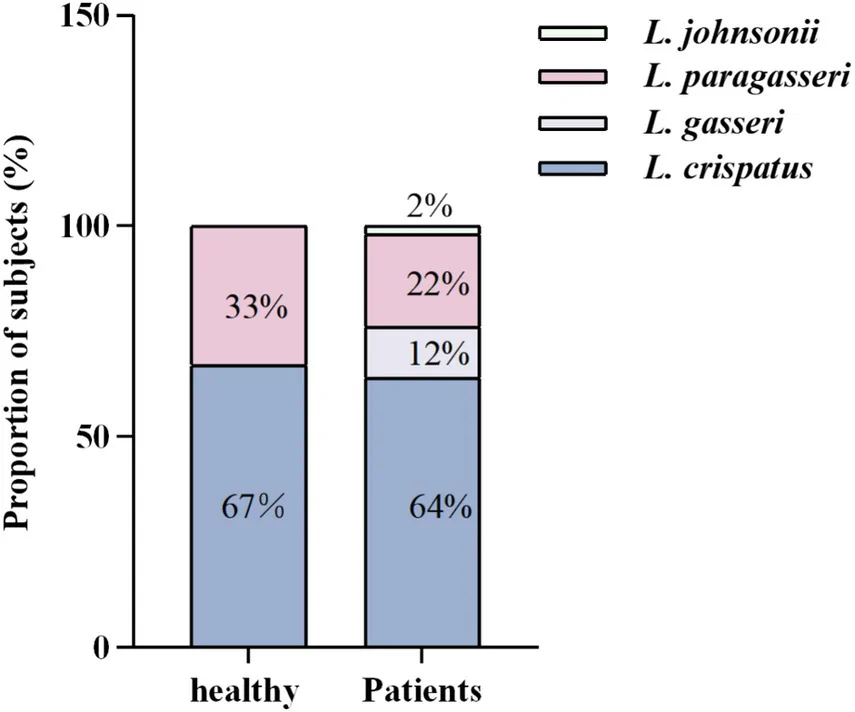
Detection rates of dominant culturable *Lactobacillus* species in healthy control groups and patients with VVC. Stacked bar chart showing the proportion of subjects whose dominant cultivable *Lactobacillus* isolate belonged to each species. Percentages were calculated as the number of subjects with a given species divided by the total number of subjects in that group. Fisher’s exact test was used for group comparisons. *L. crispatus* was dominant in both groups (VVC 32/50, 64% vs. control 20/30, 67%; *p* = 0.81). *L. paragasseri* was detected at a lower nominal frequency in VVC than in controls (11/50, 22% vs. 10/30, 33%, *p* = 0.47), but this difference did not reach statistical significance. *L. gasseri* was detected exclusively in the VVC group (6/50, 12% vs. 0/30, *p* = 0.079), and *L. johnsonii* in a single VVC isolate (1/50, 2%), which was not tested. Because three species were compared in a single-center cross-sectional cohort, these pairwise *p*-values are unadjusted and descriptive; none remained significant after correction for multiple comparisons.

### 3.6 *Lactobacillus paragasseri* enhances the inhibitory effect of fluconazole on *C. albicans* growth

Because the screened fluconazole-resistant isolates exhibited heterogeneous phenotypes across multiple resistance-related assays, we systematically evaluated the effect of *L. paragasseri* on fluconazole-resistance in *C. albicans*. The isolates were grouped for analysis, and the effects of *L. paragasseri* treatment were assessed using spot assays, biofilm-forming capacity assays, and analysis of drug efflux pump-related gene expression.

The results demonstrated that co-incubation with *L. paragasseri* reduced fluconazole MICs by 4-fold to 8-fold across the tested isolates, with MIC values decreasing from 16, 8, 16, and 8 μg/mL to 4, 1, 4, and 2 μg/mL, respectively. Notably, the post-treatment MICs of the four isolates fell below the CLSI resistance breakpoint (≥8 μg/mL), indicating that vaginal *L. paragasseri* may restore fluconazole susceptibility among the tested subset of fluconazole-resistant *C. albicans* isolates (Table 3). The spot assay results showed that, compared with the untreated group, *L. paragasseri*-treated *C. albicans* isolates produced fewer colonies and exhibited markedly impaired growth on YPD plates supplemented with 8 μg/mL fluconazole (Figures 6A–D and Supplementary Figure 3). These findings suggest that vaginal *L. paragasseri* may enhance the fluconazole susceptibility among the tested subset of fluconazole-resistant *C. albicans* isolates.

**Table 3:** Fluconazole MICs of *C. albicans* isolates prior to and following *L. paragasseri* treatment.

Strain	MIC (μg/mL)	Treatment	MIC (μg/mL)
LAB-V-24-022	16	*+L. paragasseri*	4
LAB-V-24-025	8	*+L. paragasseri*	1
LAB-V-24-112	16	*+L. paragasseri*	4
LAB-V-24-130	8	*+L. paragasseri*	2

**Figure 6:**
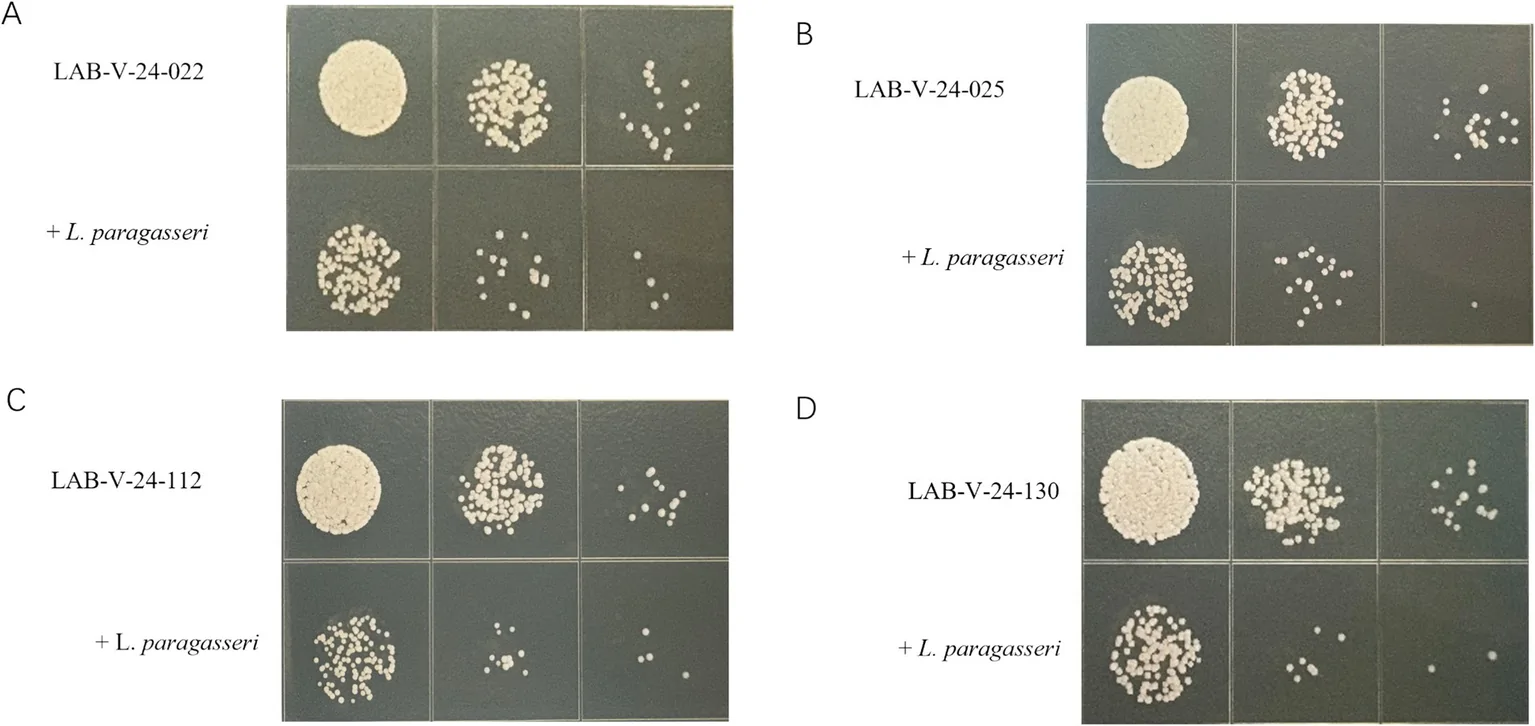
*Lactobacillus paragasseri* enhances the inhibitory effect of fluconazole on clinical isolates of *C. albicans.* **(A–D)** Representative spot-assay results for four clinical isolates of *Candida albicans* grown on solid YPD medium supplemented with 8 μg/mL fluconazole. The upper row shows the untreated *C. albicans* control group, whereas the lower row shows the group co-cultured with *L. paragasseri*. The initial concentration of the fungal suspension was 1 × 10⁵ CFU/mL, followed by three consecutive 10-fold serial dilutions spotted from left to right. Growth was assessed after 48 h of incubation. The images shown are representative of three independent experiments.

### 3.7 *Lactobacillus paragasseri* inhibits biofilm formation of *Candida albicans*

The crystal violet assay was used to quantify biofilm formation by the fluconazole-resistant *C. albicans* isolates before and after treatment with *L. paragasseri*. Absorbance was measured at 595 nm using a microplate reader. Compared with the untreated group, the *L. paragasseri*-treated group exhibited significantly lower absorbance values at 595 nm, indicating reduced biofilm biomass (Figures 7A–D). Inverted-microscopy observations further showed that the biofilm architecture changed from a dense, interconnected structure to a less-developed, yeast-cell-dominated morphology after *L. paragasseri* treatment (Figure 7E). These findings suggest that vaginal *L. paragasseri* may enhance fluconazole susceptibility by reducing biofilm formation of four clinical isolates of fluconazole-resistant *C. albicans*.

**Figure 7:**
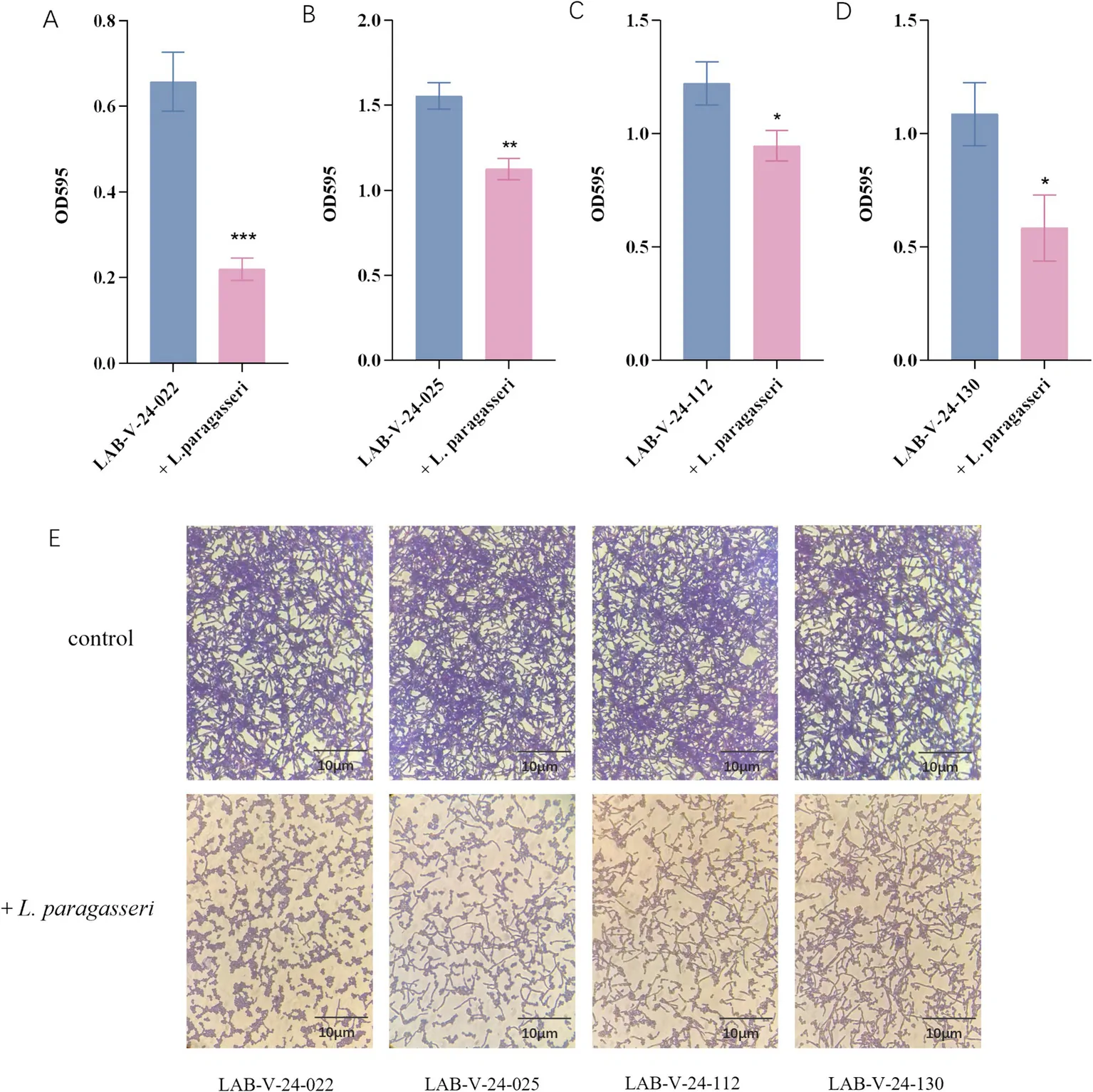
Effect of *L. paragasseri* on biofilm formation of four clinical isolates of fluconazole-resistant *C. albicans*. **(A–D)** Quantitative analysis of biofilm biomass in four *C. albicans* isolates before and after treatment with *L. paragasseri*. According to the calculated OD values, the percentages of biofilm reduction for the above four tested isolates were 66, 28, 22, and 47%, respectively. Data are presented as mean ± SD from three independent biological replicates. Statistical significance was determined by unpaired two-tailed Student’s *t*-test. *p < 0.05, **p < 0.01, ***p < 0.001. **(E)** After treatment with *L. paragasseri*, the hyphae of the four *C. albicans* isolates appeared shorter and less densely distributed, suggesting a reduction in biofilm formation. Scale bar, 10 μm. The images are representative of three independent experiments.

### 3.8 *Lactobacillus paragasseri* reduces the expression of drug efflux pump-related genes in fluconazole-resistant *C. albicans* isolates

Due to the higher expression of efflux pump genes in LAB-V-24-112 and LAB-V-24-130 and the lower expression in LAB-V-24-022 and LAB-V-24-025 during preliminary screening (Figure 3), LAB-V-24-112 and LAB-V-24-130 were selected to investigate the effects of *L. paragasseri* on efflux pump genes. RT–qPCR was used to measure the expression levels of drug efflux pump-related genes in fluconazole-resistant *C. albicans* isolates. Compared with the untreated group, treatment with *L. paragasseri* reduced the expression levels of the drug efflux pump-related genes *CDR1, CDR2*, and *MDR1* in the LAB-V-24-112 and LAB-V-24-130 isolates to varying degrees (Figures 8A–F). These findings suggest that vaginal *L. paragasseri* may enhance fluconazole susceptibility in two clinical isolates of fluconazole-resistant *C. albicans*, potentially through the reduction of drug efflux pump-related gene expression.

**Figure 8:**
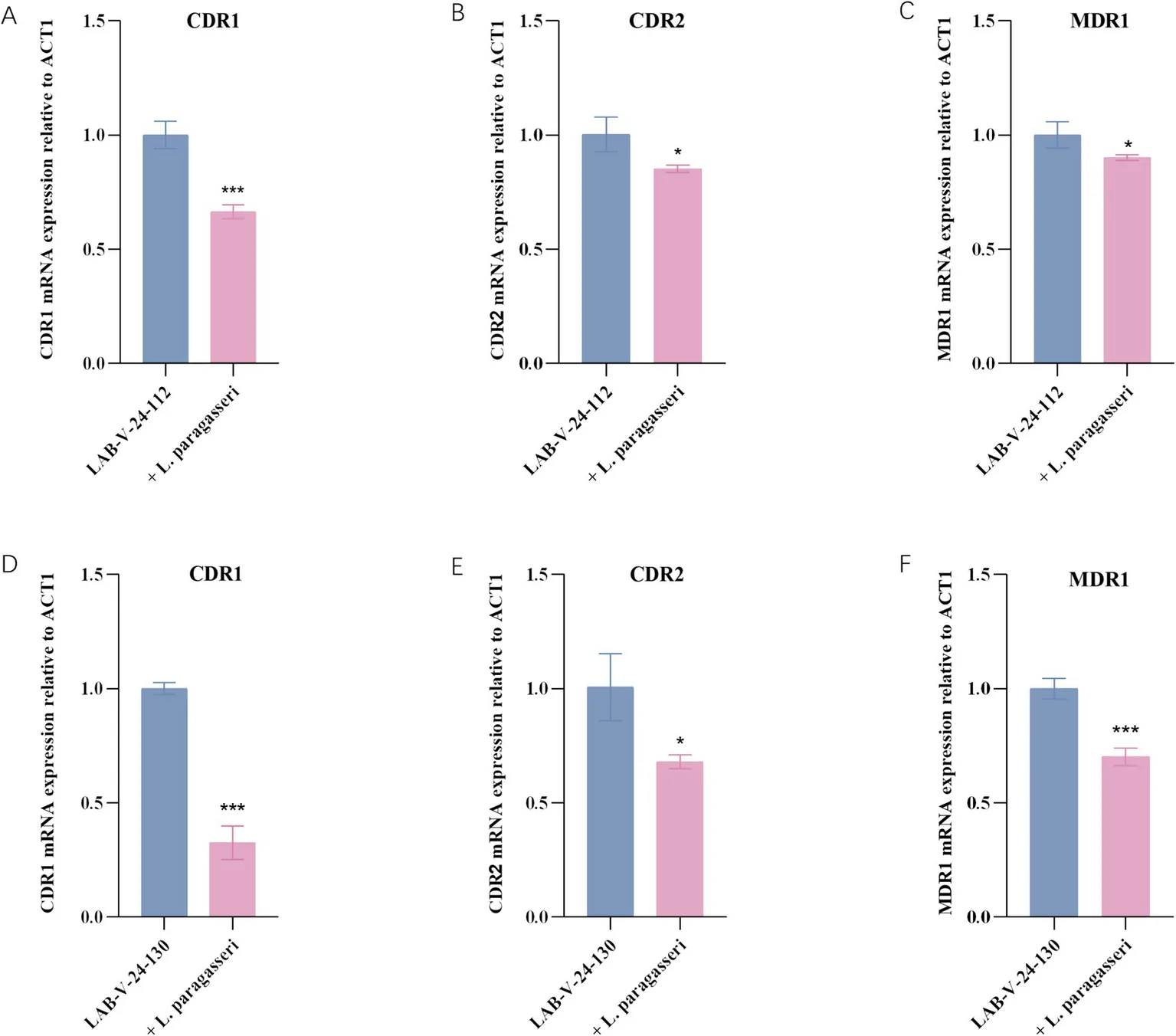
Effects of *L. paragasseri* on the expression of drug efflux pump-related genes of two clinical isolates of fluconazole-resistant *C. albicans*. **(A–C)** Following treatment with *L. paragasseri*, the expression levels of the drug efflux pump-related genes *CDR1, CDR2*, and *MDR1* were assessed in the LAB-V-24-112 isolate. In the LAB-V-24-112 isolate, *L. paragasseri* treatment reduced *CDR1* expression by 1.5-fold, *CDR2* by 1.2-fold, and *MDR1* by 1.1-fold relative to untreated controls. **(D–F)** Following treatment with *L. paragasseri*, the expression levels of *CDR1, CDR2,* and *MDR1* were assessed in the LAB-V-24-130 isolate. In the LAB-V-24-130 isolate, *L. paragasseri* treatment reduced *CDR1* expression by 3-fold, *CDR2* by 1.4-fold, and *MDR1* by 1.4-fold relative to untreated controls. The results indicate that *L. paragasseri* treatment reduces the expression of these drug efflux pump-related genes in *C. albicans*. Data are presented as mean ± SD from three independent biological replicates. Statistical significance was determined by unpaired two-tailed Student’s *t*-test. *p < 0.05, ***p < 0.001.

### 3.9 *Lactobacillus paragasseri* alleviates vaginal symptoms in a murine vaginal candidiasis model

Based on the preceding *in vitro* findings, *L. paragasseri* was shown to enhance fluconazole susceptibility in *C. albicans*, indicating its potential as an adjuvant therapy for fluconazole-resistant candidiasis. Accordingly, a murine vaginal candidiasis (VVC) model was established to investigate the *in vivo* effects of *L. paragasseri* as an adjunct to fluconazole treatment (Figure 9A).

**Figure 9:**
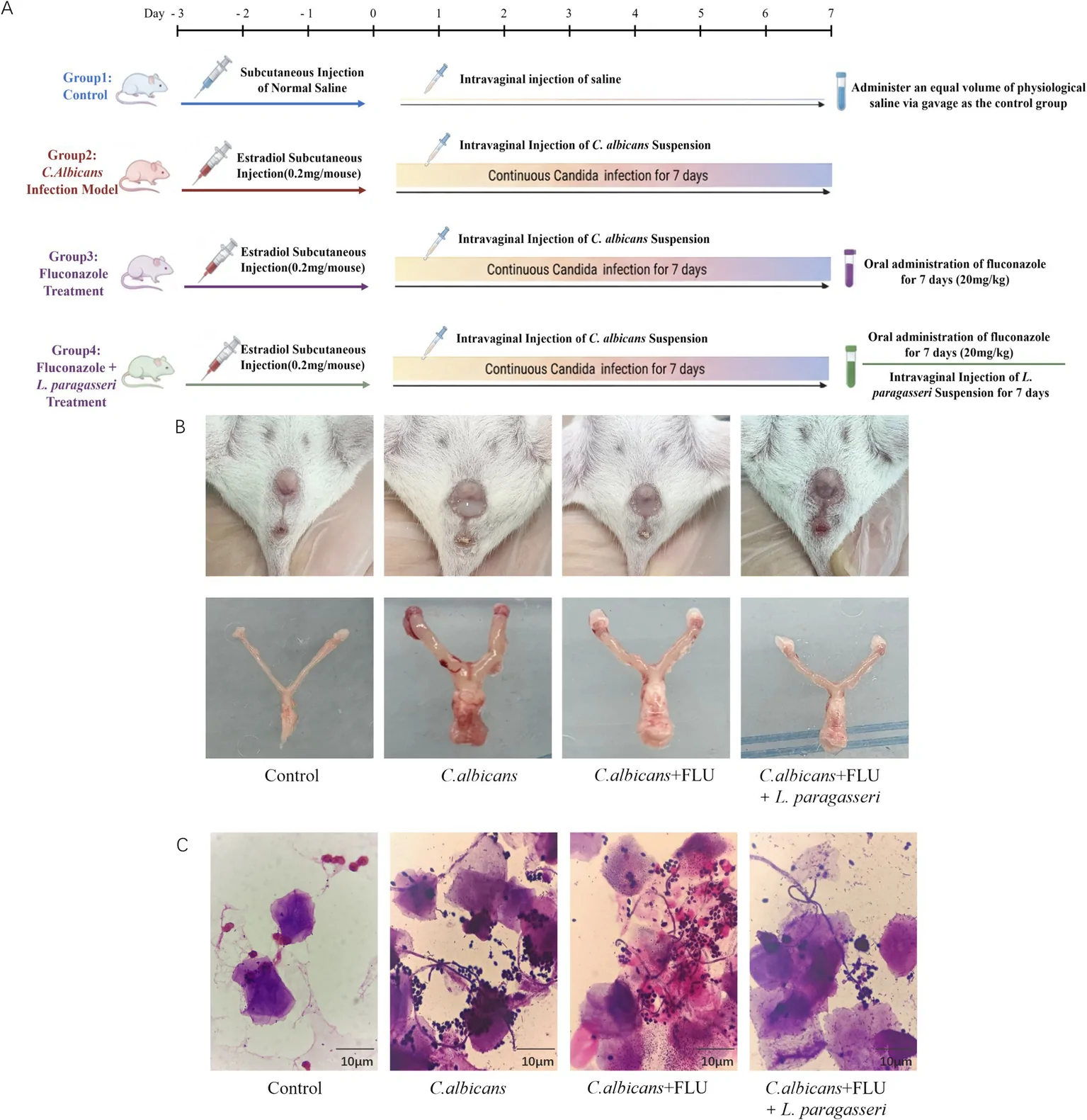
*Lactobacillus paragasseri* alleviates vaginal symptoms and reduces fungal burden in a murine vaginal candidiasis model. **(A)** Flowchart of mouse vaginal infection model. **(B)** Representative images of external vaginal signs (top) and gross reproductive anatomy (bottom) in mice from each group. **(C)** Representative images of *C. albicans* cellular morphology in vaginal lavage fluid from mice in each group. Abundant fungal cells and elongated hyphal forms were observed in both the infection model group and fluconazole monotherapy group. In contrast, after combined treatment with *L. paragasseri*, fungal burden was significantly reduced, and the morphological transition from yeast to hyphal forms was markedly inhibited. Scale bar, 10 μm. Images are representative of four independent experiments.

Mice in the healthy control group showed normal vaginal anatomy. In contrast, mice in the infection model group and fluconazole monotherapy group displayed typical VVC symptoms, including abundant white vaginal discharge, as well as erythema, edema, and enlargement of vaginal and uterine tissues. In contrast, mice receiving combined treatment with fluconazole and *L. paragasseri* showed significantly reduced VVC symptoms, suggesting that *L. paragasseri* may enhance fluconazole efficacy and reduce inflammation *in vivo* (Figure 9B). Microscopic examination of vaginal lavage fluid revealed numerous elongated hyphal forms in both the infection model group and fluconazole monotherapy group. In contrast, after combined treatment with *L. paragasseri*, the number of microorganisms was reduced, and hyphal length was significantly shortened (Figure 9C), indicating that *L. paragasseri* may inhibit the morphological transition from yeast to hyphal forms in *C. albicans*.

### 3.10 *L. paragasseri* reduces vaginal fungal burden and attenuates tissue pathology in murine vaginal candidiasis

Quantitative analysis of vaginal tissue fungal burden revealed no significant difference in *C. albicans* counts between the infection model group and fluconazole monotherapy group. In contrast, the group receiving combined treatment with *L. paragasseri* and fluconazole exhibited a significant reduction in fungal burden (Figure 10A and Supplementary Table 2). Hematoxylin and eosin (H&E) staining was performed to assess vaginal tissue pathology. In the healthy control group, the vaginal mucosa showed intact architecture with a distinct superficial keratinized layer. In contrast, the infection model group and fluconazole monotherapy group exhibited visible mucosal damage, including loss of the keratinized layer. Following combined treatment with *L. paragasseri*, mucosal damage was partially repaired, with regeneration of a thin keratinized layer observed (Figure 10B). These results indicate that *L. paragasseri*, when combined with fluconazole, may reduce *C. albicans*-induced vaginal mucosal damage by enhancing fluconazole efficacy, which contributes to the alleviation of infection symptoms. These findings suggest that *L. paragasseri* holds promise as an adjuvant probiotic therapy for fluconazole-resistant *C. albicans*-induced vaginal candidiasis. Collectively, these in-vivo data reveal the adjuvant benefit of *L. paragasseri* when combined with fluconazole against VVC. It should be noted that mouse experiments used *n* = 4 per group without *a priori* power calculation; these findings represent preliminary pre-clinical observations.

**Figure 10:**
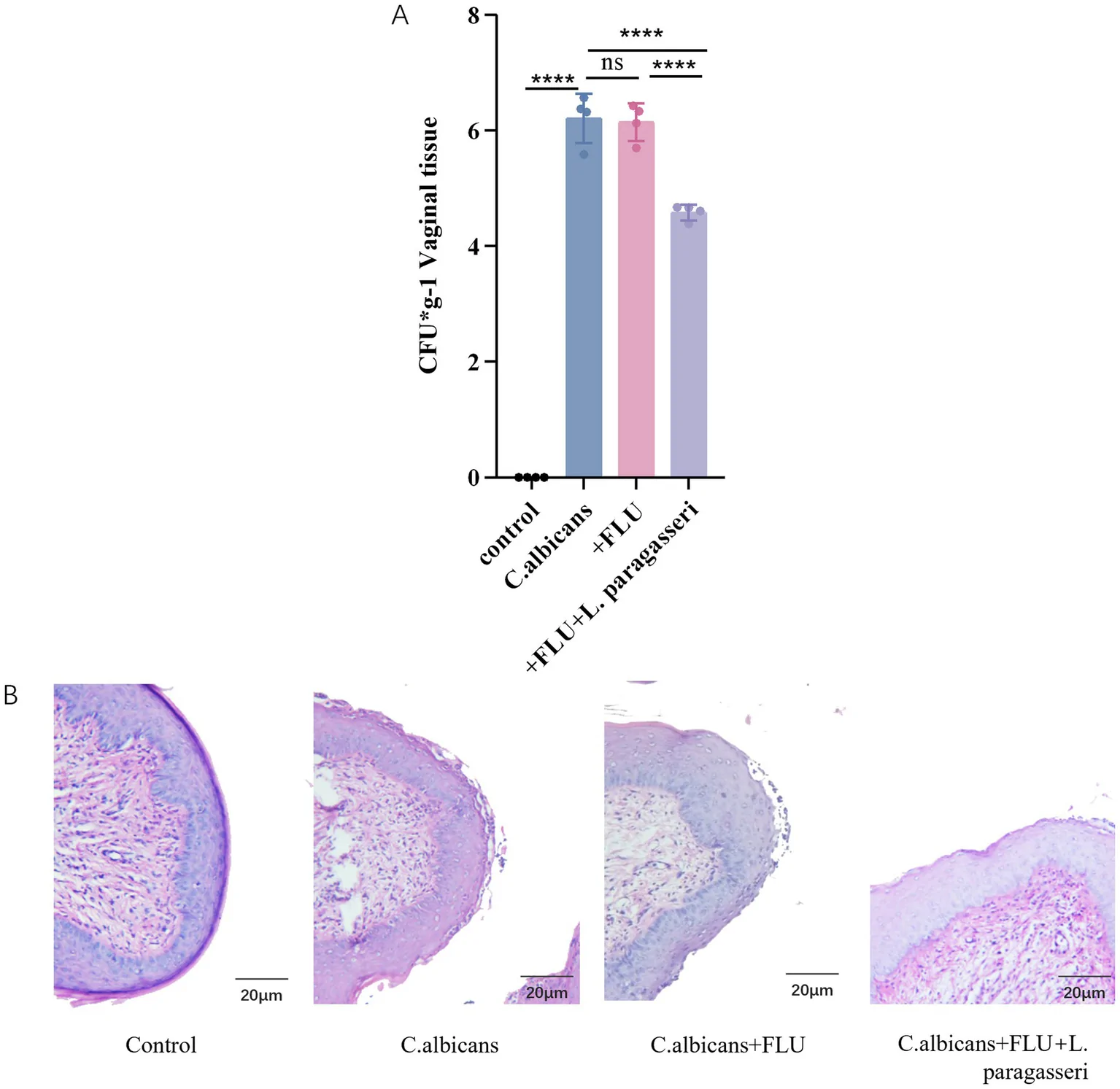
Adjunctive treatment with *L. paragasseri* reduces vaginal fungal burden and attenuates histopathological damage in murine vaginal candidiasis. **(A)** Quantitative analysis of vaginal tissue fungal burden, expressed as colony-forming units per gram of tissue (CFU/g). Data are presented as mean ± SD. *n* = 4 mice per group. Statistical significance was determined by one-way ANOVA followed by Tukey’s *post hoc* test. ns, not significant; ****p < 0.0001. **(B)** Representative hematoxylin and eosin (H&E)-stained vaginal tissue sections from mice in each group. Scale bar, 20 μm. Images are representative of three independent experiments.

## 4 Discussion

*Candida albicans* is a common opportunistic pathogen that can breach host defenses and cause vulvovaginal candidiasis (VVC) when the immune system is compromised. Currently, clinical treatment of VVC primarily involves the use of high-dose azole drugs, which has led to a rising drug resistance rate in vaginal isolates of *C. albicans*, resulting in treatment failure and disease recurrence. The mechanisms of drug resistance in *C. albicans* mainly involve overexpression of drug efflux pumps, mutations in the genes encoding drug target enzymes, and enhanced biofilm formation capacity (Teo et al., 2019; Li et al., 2023; Yan et al., 2025). In the vagina, Lactobacilli, as the dominant core microbiota, can secrete substances such as bacteriocins and lactic acid, which are crucial for maintaining the homeostasis of the vaginal microenvironment and for resisting pathogen colonization and infection (Scillato et al., 2021). Although studies have shown that certain lactobacilli can inhibit the growth of *C. albicans* (Chew et al., 2015; Wang et al., 2017). However, whether lactobacilli affect the key drug-resistant phenotypes of *C. albicans* and how they regulate them still lack systematic experimental evidence. Therefore, exploring the potential impact of symbiotic lactobacilli on the drug resistance of *C. albicans* in the vaginal microenvironment has become a critical and unresolved issue in this field.

In this study, we integrated clinical specimen isolation, phenotypic screening, and molecular mechanism analysis to identify fluconazole-resistant *C. albicans* strains and lactobacilli species associated with *C. albicans* drug resistance. First, fluconazole-resistant *C. albicans* strains were selected through a combination of drug susceptibility testing, biofilm formation assays, drug efflux pump gene expression profiling, and drug target structure analysis. These strains were subsequently used in experimental investigations.

Next, comparison of vaginal *lactobacillus* species distribution between women with VVC and healthy controls showed that *L. crispatus* was the dominant species in both groups, while *L. paragasseri* was recovered at a lower nominal frequency in VVC specimens (11/50, 22% vs. 10/30, 33%; *p* = 0.47), a difference that did not reach statistical significance. *L. gasseri* was detected only in VVC specimens (6/50, 12% vs. 0/30; *p* = 0.079). As a small, single-center, cross-sectional comparison examining three species, these distributions are descriptive and hypothesis-generating rather than confirmatory, and species-level detection should not be equated with functional outcomes given the well-documented strain-specific heterogeneity of *Lactobacillus*. Based on this directional trend, together with prior evidence that *lactobacilli* can modulate *C. albicans* resistance phenotypes, we tested the hypothesis that *L. paragasseri* may attenuate fluconazole-resistance in clinical isolates using the in-vitro and in-vivo assays below. We emphasize that this cross-sectional comparison does not stratify subjects by the antifungal susceptibility of their *Candida* isolates and therefore supports only a disease-level association with VVC, not a direct link to fluconazole resistance.

Regarding the modulation of antifungal resistance, several studies have investigated the synergistic effects of probiotics and antifungal agents. For instance, *L. rhamnosus* has been reported to enhance the inhibitory activity of fluconazole against clinically isolated fluconazole-resistant *C. albicans* strains (Dube et al., 2020). However, existing studies have primarily examined the phenotypic effects of probiotics on *C. albicans,* including changes in fungal growth, adhesion, and biofilm formation. In-depth studies are still needed to elucidate the molecular mechanisms through which probiotics modulate *C. albicans* drug resistance and to determine whether this modulation can restore fluconazole susceptibility in resistant strains and alleviate infection-associated symptoms in animal models.

The spot assay provided visual evidence that treatment with *L. paragasseri* markedly reduced the colony-forming ability of *C. albicans* in the presence of fluconazole. Biofilms are important contributors to the persistence of Candida infections and the development of antifungal resistance, as their dense structure can impede drug penetration and provide a protective microenvironment for the embedded microbial community (Ponde et al., 2021). Quantitative crystal violet staining and microscopic examination showed that *L. paragasseri* reduced biofilm formation by *C. albicans,* suggesting that it may enhance fluconazole susceptibility by compromising this resistance-associated phenotype. While our inverted microscopy observations provided clear evidence of biofilm structural changes, further studies employing confocal laser scanning microscopy or scanning electron microscopy would be valuable to visualize the three-dimensional architectural alterations in biofilms induced by *L. paragasseri*.

Recently, iminosugar compounds from *L. paragasseri* supernatant were shown to directly suppress biofilm-related gene expression without affecting quorum-sensing master regulators like *comDE*, suggesting that our observed transcriptional downregulation may be mediated by similar small-molecule effectors (Gu et al., 2022). Downregulation of the drug efflux pump genes *CDR1* and *CDR2*, which encode members of the ATP-binding cassette (ABC) transporter superfamily, and *MDR1,* which encodes a major facilitator superfamily (MFS) transporter, may reduce drug efflux and thereby increase intracellular fluconazole exposure (Lee et al., 2022). Consistent with this possibility, RT-qPCR analysis showed that *L. paragasseri* treatment downregulated the expression of *CDR1*, *CDR2*, and *MDR1* in *C. albicans*. These findings suggest that *L. paragasseri* may attenuate fluconazole-resistance by suppressing drug efflux pump expression and potentially increasing intracellular fluconazole exposure. Notably, all three Y132H mutant isolates exhibited variably elevated *ERG11* mRNA levels compared with the wild-type reference strain. This observation suggests that the Y132H substitution may not only affect protein conformation but also influence *ERG11* transcription through potential feedback or cis-regulatory mechanisms. Unlike biofilm formation and efflux pump overexpression, which represent reversible phenotypic traits, target-enzyme gene mutations constitute stable genetic alterations that are unlikely to be directly reversed by probiotic intervention. Consequently, mechanistic investigations into the ability of *L. paragasseri* to restore fluconazole susceptibility should focus on its regulatory effects on biofilm and efflux pump-related phenotypes, rather than on direct repair of *ERG11* mutations. This also implies that probiotic adjunctive therapy may be more appropriately combined with interventions targeting resistance mechanisms other than target-site mutations.

Vaginal lactobacilli, the dominant commensal bacteria in the vaginal microenvironment, play a critical role in maintaining vaginal health. Existing studies have demonstrated that probiotic therapy can effectively alleviate bacterial vaginosis (Cohen et al., 2020). To investigate the *in vivo* effect of *L. paragasseri* on *C. albicans* pathogenicity, we established a mouse vulvovaginal candidiasis (VVC) infection model using a validated protocol (Nabeta et al., 2022; Yang D. L. et al., 2018; Menon et al., 2021). Our *in vivo* results showed that *L. paragasseri* intervention significantly reduced infection-induced local inflammatory symptoms and vaginal tissue damage, decreased vaginal fungal burden, and inhibited hyphal formation in infected mice. Notably, the therapeutic efficacy of fluconazole in combination with *L. paragasseri* was superior to that of fluconazole monotherapy. *L. paragasseri* as an adjunctive treatment enhanced the antifungal effect of fluconazole. These findings provide preclinical experimental support for the development of novel resistance-reversing agents or antifungal adjuvant therapies based on *L. paragasseri,* and offer a promising approach for the adjuvant treatment of recurrent, fluconazole-resistant VVC. Collectively, this study reveals that vaginal *L. paragasseri* can modulate fluconazole-resistance in the subset of clinical fluconazole-resistant *C. albicans* isolates examined here. Within this tested strain panel, *L. paragasseri* can restore fluconazole efficacy and alleviate infection-induced pathological damage by inhibiting biofilm formation and downregulating drug-efflux-pump-related-gene expression, thereby attenuating fluconazole-resistant phenotypes. Our study cannot formally prove pharmacological synergy; the observed benefit arises from probiotic adjuvant activity, and dedicated checkerboard FICI *in vitro* assays together with an in-vivo *L. paragasseri*-only group will be required in our future work to rigorously test for true synergy. While the relatively small sample size (n = 4 per group) in the mouse experiment limits broad generalization of our *in vivo* findings, no a-priori power calculation was performed before initiating animal experiments for this exploratory study. Although statistically significant inter-group differences were detected under our experimental conditions, these murine results should be considered preliminary pre-clinical evidence. Future independent in-vivo investigations with larger group sizes will be necessary to robustly validate these observations.

Our study provides species-specific evidence that live *L. paragasseri* enhances fluconazole efficacy against resistant *C. albicans* and promotes mucosal repair, distinguishing it from prior metabolite-focused reports. However, although multiple *L. paragasseri* clinical isolates were obtained in our screening, all functional experiments were performed using only one representative strain *L. paragasseri* LPV08. In addition, inherent differences exist between murine models and the human vaginal ecosystem with respect to microbiota complexity, hormonal cyclicity, and host immunity; these factors collectively limit the generalizability of our findings. Future research could focus on isolating and identifying the active components produced by *L. paragasseri*, such as bacteriocins, organic acids, or extracellular polysaccharides. LC–MS/MS analysis of culture supernatants would be a valuable next step to directly identify the effector molecules and elucidate the precise molecular mechanisms underlying these interactions, including specific signaling-pathway regulation.

## 5 Conclusion

In the subset of clinical fluconazole-resistant *C. albicans* isolates tested in this study, *L. paragasseri* can attenuate fluconazole-resistant phenotypes through two key mechanisms: inhibition of biofilm formation and downregulation of drug efflux pump-encoding genes. Furthermore, in a murine vulvovaginal candidiasis model, adjunctive treatment with *L. paragasseri* provided adjuvant benefit when added to fluconazole therapy, reducing vaginal *C. albicans* colonization and alleviating infection-associated tissue damage. These in-vivo observations constitute preliminary pre-clinical evidence, highlighting its potential as a safe, natural adjunctive therapeutic agent for fluconazole-resistant VVC.

## Data Availability

The original contributions presented in the study are included in the article/Supplementary material, further inquiries can be directed to the corresponding authors.
