# Supplementary material for: Vaginal Lactobacillus paragasseri may attenuate fluconazole-resistant phenotypes in the subset of Candida albicans isolates and shows potential as a probiotic adjunct in antifungal therapy
Source: Front Microbiol. 2026 Oct 2;17:1951876. doi: 10.3389/fmicb.2026.1951876 (PMC13672589; doi:10.3389/fmicb.2026.1951876)
Supplement: Supplementary file 1 [file Table_1.DOCX]

Supplementary Material

**Supplementary Table 1.** Gene-Specific Primer Sequences for RT-qPCR

|  | **Primer name** | **Sequence (5’-3’)** | **Product Size(bp)** |
| --- | --- | --- | --- |
| CDR1-F | | 5’-AACCGTTTACGTTGAACACGATAT-3’ | 128bp |
| CDR1-R | | 5’-ACCAACTTCACCATCTTCAATGAC-3’ |  |
|  | |  |  |
| CDR2-F | | 5’-TGGCTAGTGTTTATATGGCAACCT-3’ | 132bp |
| CDR2-R | | 5’-AAGCTTCAGCAATTGACACTCTTT-3’ |  |
|  | |  |  |
| MDR1-F | | 5’-TCTCGGGTGGATTCTTTGCTAAT-3’ | 146bp |
| MDR1-R | | 5’-AATGGACCAAAACTAGGACCACA-3’ |  |
|  | |  |  |
| ACT1-F | | 5’-GAAGCCCAATCCAAAAGA-3’ | 130bp |
| ACT1-R | | 5’-CTTCTGGAGCAACTCTCAATTC-3’ |  |
|  | |  |  |
| ERG11-F | | 5′-TTTGACCGTTCATTTGCTCA-3′ | 110bp |
| ERG11-R | | 5′-GCAGCATCACGTCTCCAATAA-3′ |  |

**Supplementary Figure 1.** Determination of the fluconazole susceptibility phenotype of *C. albicans* by spot assay. *C. albicans* suspensions were adjusted to 1 × 10⁵ CFU/mL, followed by three successive 10-fold serial dilutions. The undiluted suspensions and serial dilutions were spotted from left to right onto YPD agar plates containing fluconazole (8 μg/mL) and incubated at 35°C for 48 h

**Supplementary Figure 2.**Mutation sites of amino acids encoded by the ERG11 gene in strains LAB-V-24-022, LAB-V-24-025, and LAB-V-24-091

**Supplementary Figure 3.** *L. paragasseri* Enhances the Inhibitory Effect of Fluconazole on Clinical Isolates of *C. albicans.* Representative spot-assay results for four clinical isolates of Candida albicans grown on solid YPD medium supplemented with 8 μg/mL fluconazole. The upper row shows the untreated *C. albicans* control group, whereas the lower row shows the group co-cultured with *L. paragasseri*. The initial concentration of the fungal suspension was 1 × 10⁵ CFU/mL, followed by three consecutive 10-fold serial dilutions spotted from left to right. Growth was assessed after 48 h of incubation.

**Supplementary Table 2.** Quantification of vaginal fungal burden in each group in the murine VVC model.

| **Group** | **control** | ***C. albicans*** | **+ FLU** | **+FLU+ *L. paragasseri*** |
| --- | --- | --- | --- | --- |
| **Log10CFU±SD** | 0±0 | 6.13±0.37 | 6.15±0.28 | 4.59±0.12 |
